# Periodical crossing of the laboratory population with the natural population would improve fitness in *S. sunia* ( *Lepidoptera: Noctuidae*)

**DOI:** 10.12688/f1000research.129183.2

**Published:** 2023-08-31

**Authors:** C. I. Real-Baca, C. A. Zuniga-Gonzalez

**Affiliations:** 1 Center for the production and reproduction of biological controllers, National Autonomous University of Nicaragua, Leon, 21000, Nicaragua; 2 Bioeconomy and Climate Change Research Center, National Autonomous University of Nicaragua, Leon, 21000, Nicaragua

**Keywords:** Noctuids, Insects, Diet, Treatments, Trials

## Abstract

**Background:** The research focused on evaluating the biological and reproductive parameters of the species *Spodoptera sunia* with the introduction of field genetic material, in the Noctuid Insect Breeding Laboratory.

**Methods:** The study was pre-experimental using three treatments with 30 individuals and three repetitions. The individuals were collected from the field, transferred to the laboratory under semi-controlled conditions of temperature and humidity, later they were quarantined for up to three generations for the assembly of the test where the crossing was carried out. In the measurement of the biological and reproductive parameters.

**Results:** The results of the treatments showed that the biological and reproductive parameters in relation to the number of pupae were T _2_ 34 males and 26 females, T _3_ was 33 males, and 27 females, T _1_ obtained 27 males and 33 females. The average weight in female T _1_ was 0.2112 mg and T _2_ was 0.2401 mg. The number of eggs in T _1_ in nine days oviposited 196 egg masses, in T _2_ in seven days 59 egg masses were oviposited, and in T _3_ 160 egg masses were oviposited. In the length parameter in mm T _3_ obtained 30 mm in larval development, T _1_ and T _2_ obtained 27 mm. Finally, in the development stages, the number of days was for T _1_ and T _2_, 24 days and for T _3_ 18 days. In the adult stages T _1_ and T _2_ it was 12 days and for T _3_ 10 days. In the egg stage in the three treatments it was three days and the pupal stage was eight days.

**Conclusions:** It is concluded that T _2_ and T _3_ presented the most optimal results. It is recommended to introduce genetic material every six months to maintain a good production of larvae of the species under study in laboratories for the production and reproduction of insect breeding.

## Introduction

In agricultural production, producers have been facing pest control, in particular, those with greater adaptability, standing out among them phytophagous insects that are difficult to combat ( Saldamando and Marquez, 2012; Brunetti *et al*., 2022; Pérez *et al*., 2003; Extremera *et al.*, 2004; Armstrong, 1994), state that the genus *Spodoptera* sp., is found within the Noctuidae family, geographically located in North America and Latin America, France, Italy, Black Sea, Balkans, England, North and Southern Africa, the Middle East, the Iberian Peninsula, and Germany ( Kergoat *et al*., 2021; Zelaya *et al*., 2022).

Due to its omnivorous nature, *S. sunia* is widespread and affects various crops. Ministry of Agriculture, Livestock, Rural Development, Fisheries and Food ( SAGARPA, 2002) mentions in the case of Mexico that *S. sunia* is considered a pest of economic importance that periodically affects essential crops. To control this pest, many laboratories produce and reproduce these organisms in order to reduce and control the population of pests in crops, some genera are *Trichogramma*, *Spalangia Telenomus*, *Cochliomyia*, *Ceratitis*, *Baculovirus* and *Nomuraea rileyi* (predators and parasitoids, viruses and entomopathogenic fungi) ( Jiménez, 2022; Badii & Abreu, 2006).

In the National Autonomous University of Nicaragua, Leon (UNAN-Leon), the Insect Breeding Laboratory began the reproduction of insects in the 80s, establishing quality controls in breeding, in such a way that the life tables allow determining the biological parameters and reproduction of the insect’s species. The subject of study becomes important given the need to study these parameters and the performance of the species *S. sunia* with the introduction of new natural genetic material (cross-species) to improve breeding and maintain optimal parameters ( Raj *et al.*, 2022; Rizo, 1994; Rizo *et al.,* 1994; Passoa, 1991). In addition, because of their importance in which these insects are host for the product of nuclear polyhedrosis virus (NPV), which is considered a biological controller of plague insects in crops of economic importance, and it avoids contaminating the environment, human health and other vertebrates ( Romero Gutierrez and Cruz Reyes, 2011; Haase *et al.,* 2015; Carrillo *et al*., 2003; Muentes, 2000).

The study focused on characterizing the biological parameters of the species *S. sunia* with the introduction of field genetic material (cross-species) at the Noctuid Insect Breeding Laboratory, Biological Controller Production and Reproduction Research Center (CIRCB), UNAN-León.

## Methods

The research was carried out at the Noctuid Insect Breeding Laboratory (CIRCB), UNAN-León Agricultural Campus, coordinates (12.11743, -86.23600). The environmental conditions were maintained under temperatures between 25°C and 27°C and a relative humidity of 60%.

The type of research was experimental, the effect of crossing specimens of the species *S. sunia* was characterized in order to strengthen the genetic material of the breeding of *S. sunia* insects used as a host in the production of the NPV ( Vásquez *et al.,* 2002; Salinas-Sánchez *et al.,* 2020).

### Laboratory protocol

The protocol used began with the collection of *S. sunia* in the field. The Sébaco area located in the coordinates (12.87845, -86.09147) was selected. The full protocol can be found on protocols.io ( Real-Baca and Zuniga-Gonzalez, 2022a). The selection criteria was that it is an area that produces vegetables and where the presence of noctuid insects is more representative, just as the producers in the area demand NPV products for the control of pest insects. After the collection, they were stored in the Noctuid Insect Breeding Laboratory (CIRCB), a quarantine period of up to three generations was spent. The soybean-based diet was prepared. This diet went through a sterilization process, and they supplemented the ingredients with vitamins and antibiotics.

In the assembly of the bioassays, two stages were considered (adults and larval length). The treatments were: T _1_: *S. sunia* species brought from the field, T _2_: *S. sunia* species from the Noctuid Insect Breeding Laboratory (CIRCB), T _3_: *S. sunia* species Crossing (field-laboratory).

250 field larvae were collected, which were subjected to a quarantine process for three generations, this to eliminate any contaminants from the field. Of these 250 larvae, a mortality of 75 larvae (30%) was obtained. This mortality was due to bacterial infection. Of the remaining 175, 10 had a poor pupal formation and 25 failed to exit the capsule, therefore, they did not reach the adult stage, leaving 140 adults at the end. After passing the three generations, approximately 200 pupae were obtained, of which 60 pupae were taken for the Field (T _1_) and Laboratory (T _2_) treatments, which were sexed and weighed to start the assembly of the test. Treatments were as follows:

• Treatment 1: *S. sunia* species brought from the field
• Treatment 2: Species of *S. sunia* from the Breeding Laboratory
• Treatment 3: *S. sunia* species Crossing (field-laboratory)

A total of 120 larvae per treatment were selected, 40 in each repetition for a total of 360 larvae in total. A six-day-old larva was deposited per four-ounce cup with a 2-cm square piece of diet, with the help of a tweezers the larvae were placed on the millimeter sheet to measure the length of each larva, and they were weighed daily on a Denver Instrument Co Serial No 3570 Analytical Balance. Also, the number of males and females per egg laying cage (replication) was considered. The following variables were evaluated:

• Larvae length: Each larva was placed on a millimeter sheet and the length measured in mm was observed.
• Hatching percentage: The number of eggs that hatched was counted visually.
• Weight of larvae: Larvae were weighed on a Denver Instrument Co Denver Analytical Balance, Cool, Serial No 3570.
• Weight of pupae: The pupae were weighed on a Denver Instrument Co Denver Analytical Balance, Cool, Serial No 3570.
• Sex of pupae: The abdominal part of the pupae was observed with the help of a Westover Scientific stereoscope ( Rizo *et al.*, 1994).
• Life cycle: The days it took in each of the larval stages were counted.

### Statistical validation

The data processing was done with the software IBM SPSS Statistics (RRID:SCR_016479) v.21 and the Student's t-test, Kolmogorov-Smirnov test (K-S), Shapiro–Wilk test (W) (normality tests), Mann–Whitney U test and Lilliefors test ( Shapiro and Wilk, 1965; Sánchez Turcios, 2015; Torres *et al*., 2012).

Student's t-test was applied considering the population studied to verify if it follows a normal distribution ( Raju, 2005; Malais and Ravensberg, 1992).

K-S test was used to verify the normality of the distribution between treatments (bioassays), equation 1 ( Massey, 1951).

Fn=x=1n∑i=1n10siyi≤x,alternative (1)

The W test was used to contrast the normality of a data set of the combined treatments, equation 2 ( Shapiro, 1965).

$$W=\frac{{\left(\sum_{i=1}^{n}a_{i}x_{\left(i\right)}\right)}^{2}}{{\left(\left(\sum_{i=1}^{n}x_{i}-\hat{x}\right)\right)}^{2}}\;$$ (2)

Mann–Whitney U test was used to check the heterogeneity of two ordinal treatments, equation 3. The starting point is:

Treatment data is autonomous.

The data are ordinal or continuous variables.

Under H _0_, the initial distribution is the same for both treatments: P(T _1_ > T _2_) = P(T _2_ > T _1_)

In the first H _1_, the value of one type of treatment tends to exceed the other: P(T _1_ > T _2_) + 0.5 P(T _1_ = T _2_) > 0.5.

$$\begin{array}{c} U_{1}=T_{1}T_{2}+\frac{T_{1}\left(T_{2}+1\right)}{2}-R_{1}\\ U_{2}=T_{1}T_{2}+\frac{T_{2}\left(T_{2}+1\right)}{2}-R_{2} \end{array}$$ (3)

Where T _1_ and T _2_ are the sizes of each treatment; R _1_ and R _2_ are the rank sum of bioassay 1 and 2 observations, respectively (the sum of the relative position of each individual in the treatment). The U statistic is defined as the minimum of U _1_ and U _2_ ( Turcios, 2015).

Lilliefors test is a normality test based on the K-S test. It was used to test H _0_ that the data come from a normally distributed population ( Lilliefors, 1967, 1969).

Table 1 shows the descriptive statistics of the data used at the time of establishing the bioassays.

**Table 1.** Descriptive statistics for the final weight (mg) of the treatments.

Treatment bio assay	N	Mean	Standard deviation	Mean standard error
T _1_	60	0.213373	0.0369291	0.0047675
T _2_	60	0.230943	0.04807	0.0062058
T _3_	60	0.204317	0.0382613	0.0049395

Source: Author’s Self calculation.

## Results and discussion

The research set out to study the biological and reproductive characteristics of *S. siuna* ( Malais and Ravensberg, 1992), known as ( *Guen*) = ( *Xylomige ssunia*) called cutworm, tiger worm, leathery worm. The characterized variables were length and weight of larvae, hatching percentage, weight and sex of pupae ( Parra *et al.,* 2022; Peterson *et al.,* 2022; Barcellos, 2022; Machado *et al*., 2020).

### Length and weight of larvae

**Figure 1.**
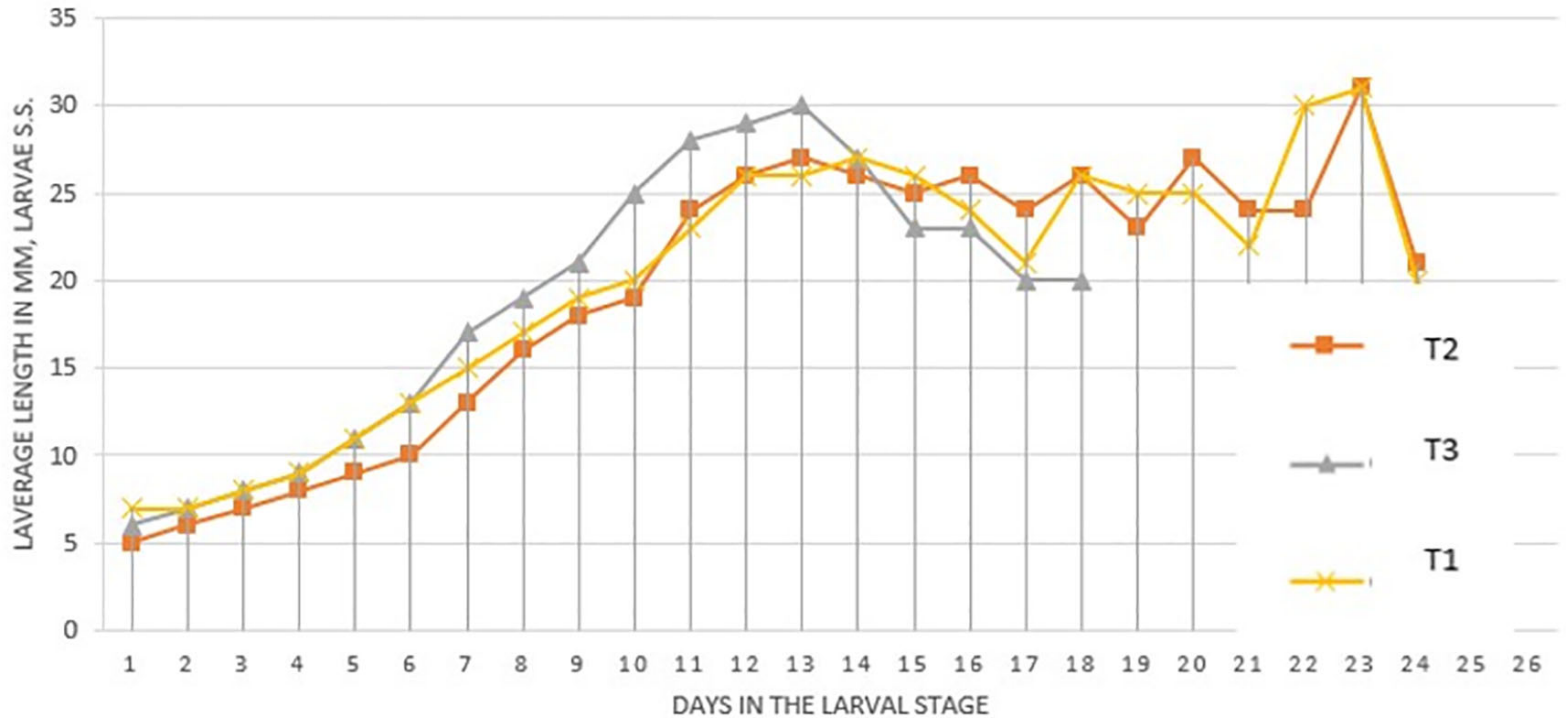
Average length (mm) of *S. sunia* larvae in the different treatments.

Figure 1 ( Real-Baca and Zuniga-Gonzalez, 2022b) presents the length (mm) of the larvae in the different treatments. It was observed that the treatments begin to have changes in their length after day 10. The three treatments reached their greatest length at 13 days, it was noted that T _3_ reached the greatest length (30 mm).

The normality of the data of the length (mm) and weight (mg) of the larvae was examined with the K-S test, the significance was 0.000 less than 0.05, so H _0_ is rejected because the data follow a normal distribution ( Table 2), for the different combinations of treatments (T _1_-T _2_ and T _1_-T _3_), it proceeded to calculate the Mann–Whitney U non-parametric test of the treatments (T _2_-T _3_, and T _3_-T _1_). In the Mann–Whitney U test, it was obtained that the significance of the treatments T _2_-T _3_, and T _3_-T _1_ was 0.000 less than 0.05, so the H _0_ is rejected, that is, there is a significant difference in terms of the length of the larvae, in the case of treatments T _2_-T _3_ the significance was 0.057 greater than 0.05, in terms of larval weight the significance was 0.098 greater than 0.05, so we accept the H _0_ of equality between the treatments, that is that there is no significant difference in terms of the length and weight of the larvae ( Table 3).

**Table 2.** Data normality test with the Kolmogorov-Smirnov statistic for length (mm) and weight (mg) during the larval stage of the treatments.

Normality test							
Variables	Treatments	Kolmogorov-Smirnov ^a^			Shapiro-Wilk		
		Statistical	gl	Sig.	Statistical	Gl	Sig.
Length in mm	Combination T _1_-T _2_	0.16	1,500	0	0.911	1,500	0
Weight in mg		0.425	1,500	0	0.082	1,500	0
Length in mm	Combination T _1_-T _3_	0.13	1,320	0	0.935	1,320	0
Weight in mg		0.417	1,320	0	0.088	1,320	0

Source: Author’s Self calculation.

^a^ Lilliefors Significance correction.

**Table 3.** Mann-Whitney U test for length (mm) and weight (mg) during the larval stage for treatments.

Statistical test ^a^			
Statistical test	Treatments	Length in mm	Weight in mg
Mann-Whitney	Combination T _2_-T _3_	265438.5	267508
Wilcoxon		547063.5	549133
Z		-1.907	-1.656
Sig. asymptotic (bilateral)		0.057	0.098
Mann-Whitney	Combination T _3_-T _1_	172098	169339
Wilcoxon		453723	450964
Z		-6.111	-6.51
Sig. asymptotic (bilateral)		0	0

Source: Author’s Self calculation.

^a^ Grouping variable: Treatments.

After day 13 in all treatments a decrease in length was observed because in that week from day 16 to day 22 the environmental conditions were not appropriate. Treatments T _2_-T _3_ reached their greatest length on day 23 with 31 mm and later descended their length to enter the pre-pupa instar.

The best larval development was in the T _3_ treatment with 30 mm because this treatment contains genes from Laboratory larvae, being more adapted to the artificial Soya diet. In the Laboratory treatment, larvae with 27 mm are obtained, this is because it is adapted to the conditions and food, continuing the T _3_ larvae with 27 mm because it is not adapted to these controlled conditions, but to natural conditions.

Romero Gutierrez and Cruz Reyes (2011) obtained an average length in the first instar of 0.5 cm, reaching its maximum length at approximately 14 days with a length of 2.5 cm (25 mm), this confirms the results obtained with a length of 27 mm for T _1_ and T _2_ treatment, the T _3_ treatment reached a length of 30 mm, this indicates a genetic improvement when introducing field material and mixing it with the laboratory material. For the T _3_ treatment, its larval growth ends at 18 days, therefore, its larval development was faster.

**Figure 2.**
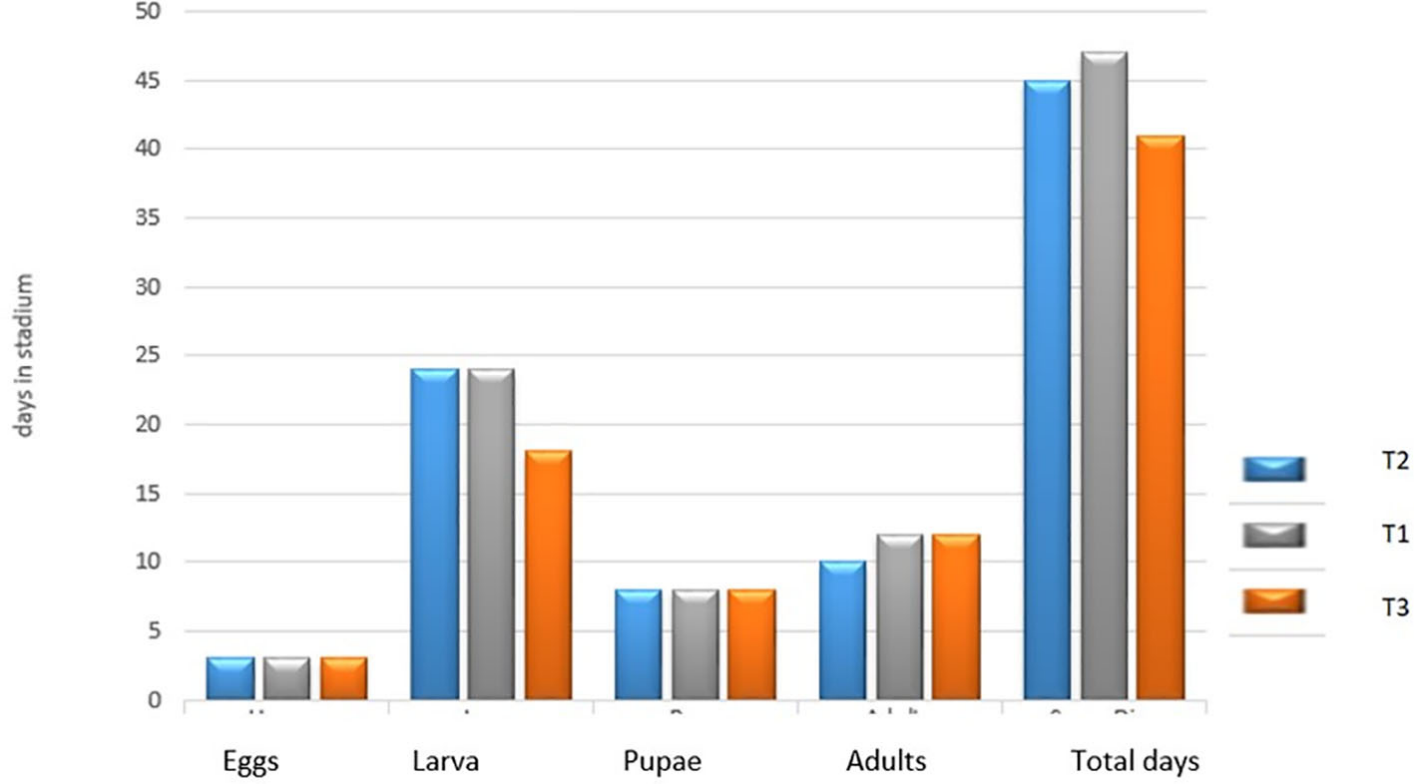
Average weight (mg) of *S. sunia* larvae of the treatments.

Figure 2 presents the weight of the larvae (mg). Treatment T _3_ on day 18 reached its highest weight (0.419 mg), this treatment being the one that reached the highest weight, then began to lose weight to enter the pre-pupa stage. The T _1_ treatment reaches a weight of 0.327 mg at 19 days and the T _2_ treatment a weight of 0.3018 mg at 20 days.

Romero Gutierrez and Cruz Reyes (2011) obtained weights of 0.25 gr (0.250 mg) and in our study it was 0.327 mg in the T _1_ and 0.301 mg for T _2_, in the case of T _3_ it was 0.429 mg. Thus, confirming that there is a difference in the crossover and field treatment since they obtained a greater weight; as soon as the laboratory treatment was similar to the weight of its study, this is due to the genetic deterioration found in the offspring.

### Hatching percentage

Table 4 and Table 5 show the results of the number of eggs in the different treatments. In the T _1_ treatment they oviposited nine days, obtaining a total of 23,354 eggs that corresponds approximately between 10 to 450 eggs for each mass with a total of 196 egg masses. The T _2_ treatment oviposited seven days for a total of 4,456 eggs ranging from four to 400 eggs each mass with a total of 59 egg masses, and the T _3_ treatment obtained the highest number of eggs, 18,025 eggs with nine days of oviposition with a number ranging from 15 to 450 eggs per egg mass for a total of 160 egg masses.

**Table 4.** Egg laying of *S. sunia* by treatment.

Day	T _1_	T _2_	T _3_
1			
2			
3	1,744		1,186
4	2,972		2,903
5	5,670		3,416
6	4,439	373	3,124
7	2,689	633	2,442
8	2,670	1,035	2,585
9	2,703	1,392	1,482
10	665	749	886
11	2	257	1
12	0	17	0
13		0	

Source: Author’s Self calculation.

**Table 5.** Oviposition range by mass size for each treatment (%).

	Size of the mass		
	Big mass (%)	Medium mass (%)	Small mass (%)
T _1_/Oviposition range			
2-42 eggs	0	0	0.11
43-82 eggs	0	0	0.89
83-122 eggs	0	0.2	0.00
123-162 eggs	0	0.7	0.00
163-202 eggs	0	0.1	0.00
203-242 eggs	0.03	0.0	0.00
243-282 eggs	0.18	0.0	0.00
283-322 eggs	0.62	0.0	0.00
323-362 eggs	0.18	0.0	0.00
T _2_/Oviposition range			
2-42 eggs	0	0	0.75
43-82 eggs	0	0	0.25
83-122 eggs	0	0.71	0
123-162 eggs	0	0.29	0
203-242 eggs	0.29	0.00	0
283-322 eggs	0.29	0.00	0
323-362 eggs	0.29	0.00	0
363-400 eggs	0.14	0.00	0
T _3_/Oviposition range			
2-42 eggs	0	0	0.31
43-82 eggs	0	0	0.27
83-122 eggs	0	0.19	0
123-162 eggs	0	0.78	0
163-202 eggs	0	0.03	0
243-282 eggs	0.48	0	0
283-322 eggs	0.10	0	0
323-362 eggs	0.19	0	0
363-400 eggs	0.23	0	0

Source: Author’s Self calculation.

In the T _3_ and T _1_ treatment, oviposition began after three days, but not in the T _2_ treatment, this was due to the fact that the fertility of the adult drops due to the fact that they are crosses between the same family, as inbreeding increases, insects decrease their performance ( Martinez, 1994; Bárcenas, 1997). The most used variables are fecundity, egg viability, development time, body size, survival and efficiency ( Huettel, 1976; Moore *et al.,* 1985; Smith *et al.,* 1996). This confirms that it is important to introduce field material to the laboratory to obtain a better fertility and thus obtain a better reproduction in the breeding of insects.

The normality of the data for the final weight in mg was done with the K-S test, the significance was 0.200 greater than 0.05, so H _0_ was accepted, likewise the data follow a normal distribution ( Table 6), for the different combinations of treatments, the parametric Student’s T-test was calculated for independent samples of the treatments (T _2_-T _3_, T _3_-T _1_, T _2_-T _1_). It was observed that the homogeneity of variance is fulfilled because the significance is greater than 0.05. In the Student’s T-test, a significance of 0.027 less than 0.05 was obtained, it was concluded that there is a significant difference between the final weight in mg of *S. sunia* of T _2_ and T _1_ ( Table 7).

**Table 6.** Normality test for weight (mg).

Treatment combinations	Kolmogorov-Smirnov ^a^			Shapiro-Wilk		
	Statistical	Gl	Sig.	Statistical	gl	Sig.
T _2_-T _1_	0.044	120	.200 ^*^	0.974	120	0.021
T _3_-T _1_	0.059	120	.200 ^*^	0.983	120	0.135
T _2_-T _1_	0.048	120	.200 ^*^	0.974	120	0.019

Source: Author’s Self calculation.

* This is a lower limit of the true significance.

^a^ Lilliefors significance correction.

**Table 7.** Independent samples test for weight (mg).

Treatment combinations	Variance	Levene's test of equality of variances		Student's T-test for equality of means					95% confidence interval of the difference	
		F	Sig.	t	gl	Sig. (bilateral)	Difference of means	Difference of means
									Lower	Higher
T _2_-T _3_	*σ	1.627	0.205	3.357	118	0.001	0.0266267	0.0079316	0.0109199	0.0423335
	**σ			3.357	112.346	0.001	0.0266267	0.0079316	0.0109117	0.0423417
T _3_-T _1_	*σ	0.15	0.7	-1.319	118	0.19	-0.0090567	0.006865	-0.0226512	0.0045379
	**σ			-1.319	117.852	0.19	-0.0090567	0.006865	-0.0226514	0.0045381
T _2_-T _1_	*σ	0.15	0.7	-1.319	118	0.19	-0.0090567	0.006865	-0.0226512	0.0045379
	**σ			-1.319	117.852	0.19	-0.0090567	0.006865	-0.0226514	0.0045381

Source: Author’s Self calculation.

* Equal variances are assumed.

** Equal variances are not assumed.

In the Student’s T-test, a significance of 0.001 less than 0.05 was obtained, it was concluded that there is a significant difference between the final weight in mg of *S. sunia* in the Laboratory treatment and the Crossing. In the Student’s T-test, a significance of 0.190 greater than 0.05 was obtained, it was concluded that there is no significant difference between the final weight in mg of *S. sunia* of T _3_ and T _1_.

### Weight and sex of pupae

Table 8 shows the number of pupae of the populations of *S. sunia* from treatment T _2_, which obtained a total of 60 pupae, of which 34 are male pupae and 26 female pupae, for a sex ratio of 0.7:1. Treatment T _3_ obtained a total of 60 pupae, 28 females and 32 males for a sex ratio of 0.8:1 and in treatment T _3_ a total of 60 pupae, 33 females and 27 males, and a sex ratio of 1.2 were obtained: 1. This indicates that the proportion for T _2_ and T _3_ was not optimal and that of T _3_ was the most optimal, this is favorable considering that more female pupae than male pupae are needed in the laboratory for greater reproduction and better conditions.

**Table 8.** Average weight in (mg) and sex of final pupae in the *Spodoptera sunia* treatments.

Average weight and sex of initial pupae				
Treatments	Number of pupae		Weight (mg)	
	Females	Males	Females	Males
T _1_	28	32	0.2112	0.1983
T _2_	26	34	0.2401	0.2239
T _3_	33	27	0.2197	0.2057

Source: Author’s Self calculation.

With respect to weight, we observed that the female *S. sunia* pupae in the three treatments were larger than the males. The T _2_ treatment had a higher weight (0.2401), but not the T _3_ and T _1_ treatments (0.2197 and 0.2112), respectively.

### Lifecycle

**Figure 3.**
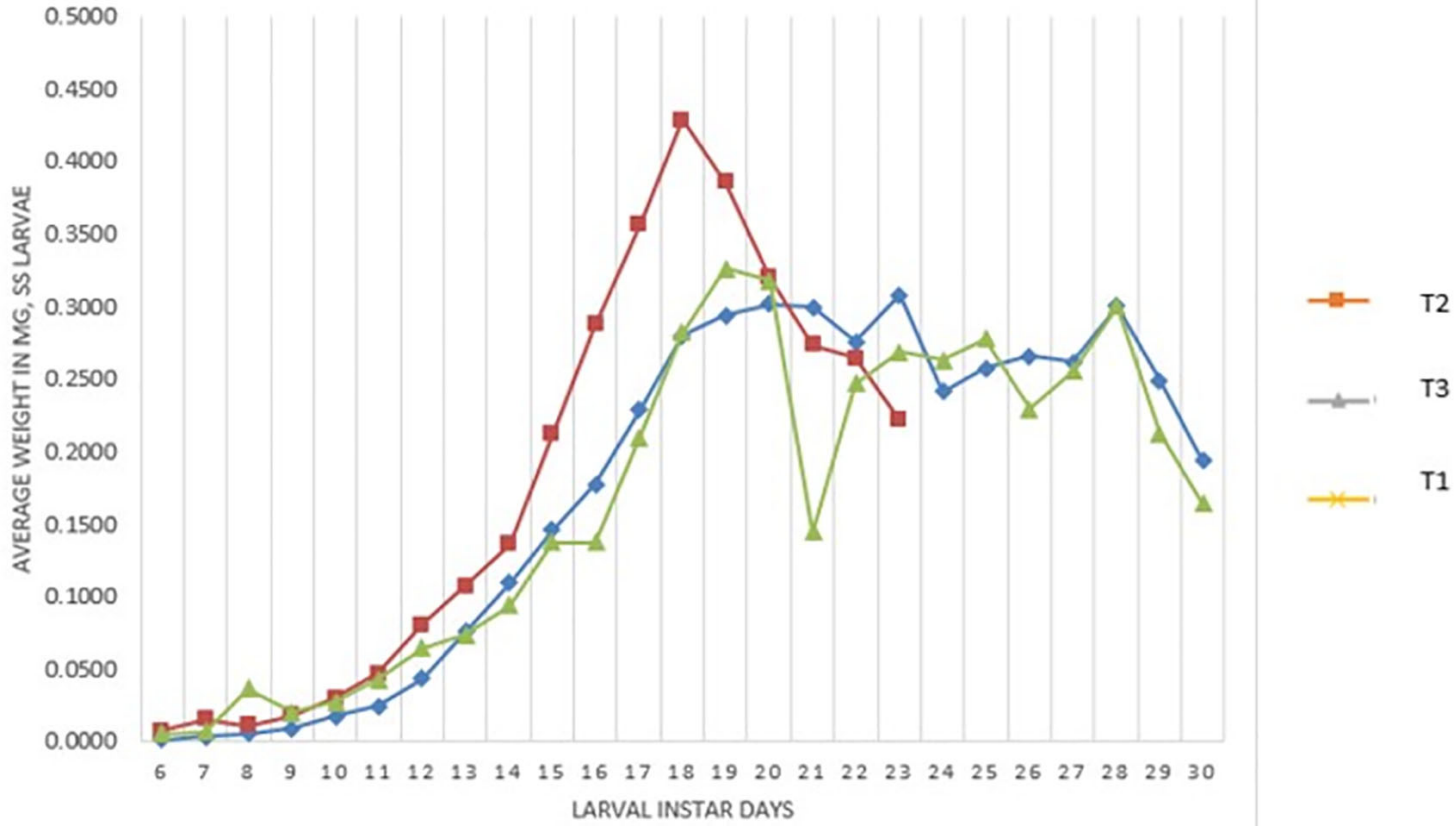
Number of days in each stage of development of *S. sunia* in the treatments.

Figure 3 presents the average number of days in each stage of development of the species *S. sunia* in the three treatments. In the egg and pupal stages, the three treatments had no difference in days. In the larval stage for treatment T _2_ and T _1_ there was no difference (24 days), but not for treatment T _3_, which presented a small difference (18 days). In the adult stage, the T _2_ treatment obtained 10 days and in the T _1_ and T _3_ treatments they obtained a difference of two days (12 days).

Romero Gutierrez and Cruz Reyes (2011) and Delgado and Hernández (2008) reported that the adult stage lasted 15.56 days. In this research for the T _2_, the adult stage lasted 10 days, while in the T _1_ and T _3_ treatment it lasted 12 days, less than what they report in their work.

Romero Gutierrez and Cruz Reyes (2011) reported that the duration of the biological cycle, from egg to pupae, was 36.16 days, which was similar to the data obtained in the UNAN-León laboratory under controlled conditions and fed with an artificial soy-based diet. As can be seen, there is an average difference approximately for the T _3_ treatment of five more days, for the T _1_ treatment of 11 days and for the T _2_ treatment it was nine more days. According to Romero Gutierrez and Cruz Reyes (2011), this is due to the type of feeding and conditions in which these insects are raised, which is why the days of their biological cycle increase.

## Conclusions

The treatment combinations were statistically validated. Based on the characterization of the periodical crossing of populations *S. siuna*, it is concluded that the best results are presented in treatments T _1_ and T _3_. The considerations of the study were that the biological cycle of the species *S. sunia* brought from the Field (T _3_) lasted 47 days, that of the Laboratory (T _2_) 45 days and that of the Crossing 41 days. The highest average larval weight obtained from the species *S. sunia* obtained was T _3_ with 0.4288 mg. The best sex ratio of the species *S. sunia* was T _3_ (1.22:1). The longest treatment was T _3_ (30 mm). T _1_ obtained the highest number of eggs (1,744–5,670).

Based on these results, it is recommended to introduce genetic material every six months to maintain a good production of larvae of the species. It is also recommended to maintain stable climatic conditions (temperature and humidity) in the Insect Breeding Laboratory of the populations *S. sunia* ( Montezano *et al.,* 2014; Salinas-Sánchez *et al*., 2020). When preparing the diet, all the nutritional requirements must be met so that the reproduction of the insects of the populations *S. siuna* develops well. Finally, after moving to individual vessels, the diet is changed at least twice during the larval stage to prevent drying out and for better larval development.

## Data Availability

Figshare: Data for: Cross-species characterization in the reproduction of *S. sunia* (Lepidoptera: Noctuidae). https://doi.org/10.6084/m9.figshare.21671669 ( Real-Baca and Zuniga-Gonzalez, 2022b). This project contains the following underlying data:
‐ Data.csv ‐ Fig 1. tif ‐ Fig 2. tif ‐ Fig 3. tif ‐
Table 1. csv ‐
Table 2. csv ‐
Table 3. csv ‐
Table 4. csv ‐
Table 5. csv ‐
Table 6. csv ‐
Table 7. csv ‐
Table 8. csv Data are available under the terms of the Creative Commons Attribution 4.0 International license (CC-BY 4.0).
