# Supplementary material for: Periodical crossing of the laboratory population with the natural population would improve fitness in S. sunia ( Lepidoptera: Noctuidae)
Source: F1000Res. 2023 Nov 14;12:30. Originally published 2023 Jan 9. [Version 3] doi: 10.12688/f1000research.129183.3 (PMC11196938; doi:10.12688/f1000research.129183.3)
Supplement: Peer review history

# Reviewer report: Reviewer response for version 3

**DOI:** 10.5256/f1000research.158483.r282159

**Published:** 2024-06-24

**Authors:** Rajanikanth P

**Affiliations:** 1 Department of Entomology, College of Agriculture, Professor Jayashankar Telangana State Agriculture University, Hyderabad, Telangana, India

Concept is good

 But the very purpose of study is to help NPV production or to maintain general vigor of laboratory populations is the question?

 What ever the objective may be 

 1) Insects come up well only under natural diet 

 the artificial diet is only for our ease of mass producing the insects for a brief period of time.

 2) Though artificial diet is a balanced food it only gives required energy to insect for promoting to next stages but it does not activate the real potential of the insect which the insect derives when feeding on live food. As the plants produce certain defense molecules which the insect tries to fight back.

 3) The very problem with inbreeding is lack of immunity in the cultures and most of them die due to exposure of the cultures to their own excreta which they release in the same vicinity.

 4) The excreta released by the insects after eating the artificial diet is totally different from that released from natural diet. 

 5)These excreta attract large quantum of microbes other than entomopathogens for which the larvae are very susceptible.

 6) Hence periodical cross will always help in restoring the general vigour and enhances immunity in the insects.

 The data on these points will really help in drawing drawing perfect conclusions for the current research.

Is the work clearly and accurately presented and does it cite the current literature?

Yes

If applicable, is the statistical analysis and its interpretation appropriate?

Yes

Are all the source data underlying the results available to ensure full reproducibility?

Yes

Is the study design appropriate and is the work technically sound?

Yes

Are the conclusions drawn adequately supported by the results?

Partly

Are sufficient details of methods and analysis provided to allow replication by others?

Yes

Reviewer Expertise:

Host plant resistance, Insect nutrition, Insect Immunity and Insecticide resistance

I confirm that I have read this submission and believe that I have an appropriate level of expertise to confirm that it is of an acceptable scientific standard.

# Reviewer report: Reviewer response for version 3

**DOI:** 10.5256/f1000research.158483.r223073

**Published:** 2023-11-27

**Authors:** Hend Omar Mohamed

**Affiliations:** 1 Biological Control Research Department, Plant Protection Research Institute, Agricultural Research Center, Giza, Egypt

No further comments to make .

Is the work clearly and accurately presented and does it cite the current literature?

Yes

If applicable, is the statistical analysis and its interpretation appropriate?

Yes

Are all the source data underlying the results available to ensure full reproducibility?

Yes

Is the study design appropriate and is the work technically sound?

Yes

Are the conclusions drawn adequately supported by the results?

Yes

Are sufficient details of methods and analysis provided to allow replication by others?

Yes

Reviewer Expertise:

Biological Control, Integrated pest management and Economic Entomology.

I confirm that I have read this submission and believe that I have an appropriate level of expertise to confirm that it is of an acceptable scientific standard.

# Reviewer report: Reviewer response for version 2

**DOI:** 10.5256/f1000research.154183.r207549

**Published:** 2023-10-31

**Authors:** Florencia Vianna, Leticia Russo

**Affiliations:** 1 Instituto Spegazzini, National University of La Plata, La Plata, Buenos Aires Province, Argentina

Periodical crossing of the laboratory population with the natural population would improve fitness in *S. sunia* (Lepidoptera: Noctuidae)

 The authors have studied the biological and reproductive parameters of the species *Spodoptera sunia* (Lepidoptera: Noctuidae) in different populations (field, laboratory and crossing populations) and investigates the results of the periodic introduction of field genetic material in laboratory colonies. Based on the results, the authors recommend introducing genetic material from the field every six month, also to maintain stable climatic conditions and to provide the adequate diet that should be changed every two days during the larval stage.

**Broad comments**

- The introduction section could be expanded providing further information about the plague and explaining the importance of the investigation.
- Material and methods section should be better explained, describing properly the protocols followed.
- Results and discussion section should be rewritten. There are many mistakes in the tables and figures. Also the text and tables present redundant information.

**Specific comments**

 Title

- The title should provide the complete name of the lepidopteran species: Spodoptera Also the Order and Family should not go in italics: Lepidoptera: Noctuidae

Abstract

- Please delete “in the Noctuid Insect Breeding Laboratory”.

Introduction

- When you first mention the species *S. susinia*, please provide the complete name of the species: *Spodoptera sunia *(Guenée) (Lepidoptera: Noctuidae).
- “To control this pest, many laboratories produce and reproduce these organisms in order to reduce and control the population of pests in crops, some Genera are *Trichogramma, Spalangia Telenomus, Cochliomyia, Ceratitis, Baculovirus* and *Nomuraea rileyi* (predators and parasitoids, viruses and entomopathogenic fungi) (Jiménez, 2022; Badii & Abreu, 2006)”. Please delete the word 'this', you're referring to biocontrol agents not the plague.

Methods

- “The research was carried out at the Noctuid Insect Breeding Laboratory (CIRCB), UNAN-León Agricultural Campus, coordinates (12.11743, -86.23600)” Please delete the word coordinates.
- Laboratory protocol, first paragraph last sentence: whose they? Please provide the correct sentence.
- Page 4 first paragraph : Please do not start the sentence with a number, for example you could say “A total of 250...”
- Page 4: You've already mention which were the treatments.

​​​​​​Statistical validation

- “The data processing was done with the software IBM SPSS Statistics (RRID:SCR_016479) v.21 and the Student's t-test, Kolmogorov-Smirnov test (K-S), Shapiro–Wilk test (W) (normality tests), Mann–Whitney U test and Lilliefors test (Shapiro and Wilk, 1965; Sánchez Turcios, 2015; Torres *et al.*, 2012).” Please correct the sentence. You say and mention the test and do not finish the sentence correctly.
- Student t-test is not used to verify normality of data sets.
- There is no need to provide the formula of neither the statistical tests nor the hypothesis that were tested in material and methods.
- Table 1 should be presented in results section, and also you should check the information that provides.

Results and discussion

 Length and weight of larvae

- Figure 1: please express correctly the legend in the y axis.
- Figure 2 does not provide the information on the weight of the different stages. Please place the correct figure.
- There is no need to describe the hypotheses that were tested; you should summarize the information correctly. The information provided in table 2 is also redundant since it provides repeated information.
- Which values does table 3 provide? You should clarify and place the correct values.
- Figure 2 does not present the average weight of larvae Hatching percentage.
- Please check the values provided in table 4 and those express in the text. There are several mistakes or at least inconsistencies. If the number of eggs is an average you should also provide standard deviation values. Furthermore, strange values are observed, within the same treatment for example in T1: 1,744 and 665.

​​Weight and sex of pupae

- Figure 3, colors in the figure does not match those in the legend. Also, in the figure (x axis) you only refer to the larval stage but in the text you mention egg, larval, pupal and adult stage. Furthermore, which are the statistical values for you to say that there are or not significative differences?

Is the work clearly and accurately presented and does it cite the current literature?

Partly

If applicable, is the statistical analysis and its interpretation appropriate?

Partly

Are all the source data underlying the results available to ensure full reproducibility?

Yes

Is the study design appropriate and is the work technically sound?

Yes

Are the conclusions drawn adequately supported by the results?

Yes

Are sufficient details of methods and analysis provided to allow replication by others?

No

Reviewer Expertise:

Biological control, entomology, micology

We confirm that we have read this submission and believe that we have an appropriate level of expertise to state that we do not consider it to be of an acceptable scientific standard, for reasons outlined above.

# Response

**Published:** 2023-11-02

**Authors:** C. A. Zuniga-Gonzalez

**Affiliations:** Agroecology. ECAV. CIByCC., National Autonomous University of Nicaragua, Leon. Bioeconomy and Climate Change Research Center., Leon, Leon, Nicaragua

Reviewer 3

 Periodical crossing of the laboratory population with the natural population would improve fitness in S. sunia (Lepidoptera: Noctuidae)

 The authors have studied the biological and reproductive parameters of the species Spodoptera sunia (Lepidoptera: Noctuidae) in different populations (field, laboratory and crossing populations) and investigates the results of the periodic introduction of field genetic material in laboratory colonies. Based on the results, the authors recommend introducing genetic material from the field every six month, also to maintain stable climatic conditions and to provide the adequate diet that should be changed every two days during the larval stage.

**Response to reviewer**

 We thank reviewer 3 for his contribution and contributions to improving the quality of the manuscript. We appreciate his accurate observations in the methodology section.

 Broad comments

 The introduction section could be expanded providing further information about the plague and explaining the importance of the investigation.

**Response to reviewer**

 We appreciate your observations, in general and according to reviewers 1 and 2, a review and rewriting of this section was made, highlighting the importance and relevance of this study in countries like ours.

 Material and methods section should be better explained, describing properly the protocols followed.

 We greatly appreciate your observation. We have added in the article the main aspects of the methodology and the protocol applied. However, we have added and made available to the public the protocols.io repository (Real-Baca and Zuniga-Gonzalez, 2022a), where we indicate the protocol, and the materials used throughout the collection process, identification both in the field phase and from laboratory.

 Results and discussion section should be rewritten. There are many mistakes in the tables and figures. Also the text and tables present redundant information.

**Response to reviewer**

 We greatly appreciate your observation. Improvements were made to the tables and Figures 2 and 3 were ordered as there was a location problem (they were inverted). In the same way, we have generally reviewed the article to correct the grammar that other reviewers also indicate.

 Specific comments

 Title

 The title should provide the complete name of the lepidopteran species: Spodoptera Also the Order and Family should not go in italics: Lepidoptera: Noctuidae

 Abstract

 Please delete “in the Noctuid Insect Breeding Laboratory”.

**Response to reviewer**

 Dear reviewer has been corrected it as you indicate.

 Introduction

 When you first mention the species S. susinia, please provide the complete name of the species: Spodoptera sunia (Guenée) (Lepidoptera: Noctuidae).

  “To control this pest, many laboratories produce and reproduce these organisms in order to reduce and control the population of pests in crops, some Genera are Trichogramma, Spalangia Telenomus, Cochliomyia, Ceratitis, Baculovirus and Nomuraea rileyi (predators and parasitoids, viruses and entomopathogenic fungi) (Jiménez, 2022; Badii & Abreu, 2006)”. Please delete the word 'this', you're referring to biocontrol agents not the plague.

**Response to reviewer**

 Dear reviewer has been corrected it as you indicate.

 Methods

 “The research was carried out at the Noctuid Insect Breeding Laboratory (CIRCB), UNAN-León Agricultural Campus, coordinates (12.11743, -86.23600)” Please delete the word coordinates.

  Laboratory protocol, first paragraph last sentence: whose they? Please provide the correct sentence.

 Page 4 first paragraph : Please do not start the sentence with a number, for example you could say “A total of 250...”

 Page 4: You've already mention which were the treatments.

 ​​​​​​ Statistical validation

 “The data processing was done with the software IBM SPSS Statistics (RRID:SCR_016479) v.21 and the Student's t-test, Kolmogorov-Smirnov test (K-S), Shapiro–Wilk test (W) (normality tests), Mann–Whitney U test and Lilliefors test (Shapiro and Wilk, 1965; Sánchez Turcios, 2015; Torres et al., 2012).” Please correct the sentence. You say and mention the test and do not finish the sentence correctly.

  Student t-test is not used to verify normality of data sets.

**Response to reviewer**

 Dear reviewer has been corrected it as you indicate.  The t-test was clarified and corrected.

 There is no need to provide the formula of neither the statistical tests nor the hypothesis that were tested in material and methods.

**Response to reviewer**

 Dear reviewer has been corrected it as you indicate.  We would like to keeping the formula for academic purposes.

 Table 1 should be presented in results section, and also you should check the information that provides.

**Response to reviewer**

 Dear reviewer we thanks for this observation.  We has been added it the analysis of the table, but  we consider keep in this section because is part of the data.

 Results and discussion

 Length and weight of larvae

 Figure 1: please express correctly the legend in the y axis.

 Figure 2 does not provide the information on the weight of the different stages. Please place the correct figure.

**Response to reviewer**

 Dear reviewer your observation is correct.  It was a problem of confusion. The Figure 3 is in the place of Figure 2 and vice versa.

 There is no need to describe the hypotheses that were tested; you should summarize the information correctly. The information provided in table 2 is also redundant since it provides repeated information.

  Which values does table 3 provide? You should clarify and place the correct values.

**Response to reviewer**

 Dear reviewer the comment of the table 2 was eliminated and change for other that no describe the hypothesis, regarding to table 3, we have left the comments on the comparisons of the treatments aside since you are asking about the applied values.

 Figure 2 does not present the average weight of larvae Hatching percentage.

 Please check the values provided in table 4 and those express in the text. There are several mistakes or at least inconsistencies. If the number of eggs is an average you should also provide standard deviation values. Furthermore, strange values are observed, within the same treatment for example in T1: 1,744 and 665.

**Response to reviewer**

 Dear reviewer thanks for your observations. The data was corrected is 1744  and not 1,744. Is was a problem of consider a decimal by comma.   Regarding to the Figure 2 has been corrected it

 ​​Weight and sex of pupae

 Figure 3, colors in the figure does not match those in the legend. Also, in the figure (x axis) you only refer to the larval stage but in the text you mention egg, larval, pupal and adult stage. Furthermore, which are the statistical values for you to say that there are or not significative differences?

**Response to reviewer**

 Dear reviewer has been corrected it.

 Bst Rgs

 Authors

# Reviewer report: Reviewer response for version 2

**DOI:** 10.5256/f1000research.154183.r202750

**Published:** 2023-10-31

**Authors:** SB Suby

**Affiliations:** 1 ICAR-Indian Institute of Maize Research, Ludhiana, India

When referring to a species for the first time in the title as well as in the main text we use the full genus name.

 Title: Write *Spodoptera sunia* in place of *S. sunia*

 Background *: *Write *Spodoptera sunia* in place of *S. sunia*

 The manuscript needs editing for proper use of English grammar. For instance, I am rewriting the abstract as given below:

*" **Methods**: The experiment consisted of three treatments and three repetitions involving 30 individuals each. Field-collected S. sunia population was reared in the laboratory under semi-controlled conditions of temperature and humidity for three generations. The progeny of the third generation was crossed with the laboratory population and the resulting progeny was observed for growth and reproductive fitness.*

***Results: **The results revealed that the hybrid progeny (T3) has a sex ratio (F: M) of  0.82, as against 1.22 and 0.76 observed in the field (T1) and lab populations (T2) respectively. The T1 females oviposited 196 egg masses as against 160 and 59 egg masses by T3  and T2 females respectively. The larval growth was more in hybrid progeny with shorter larval duration. However, no variation was observed in the incubation and pupal periods.*

***Conclusion:** Since the most optimal results were obtained in T2 and T3,  it is recommended to introduce genetic material every six months to maintain a good laboratory population of the insect host under study and better breeding of its natural enemies."*

 Pardon me, I can't reframe the MS beyond this point, it is a tedious job. Kindly get the rest edited for English language and grammar, preferably by a researcher.

 Also, the authors did not incorporate all the previous suggestions eg. *S. siuna* is used again in place of *S. sunia *and limiting two digits after the whole number. eg. 0.4288 mg. It should be 0.43 mg. Also am seeing the same wrongly placed graphs.

Is the work clearly and accurately presented and does it cite the current literature?

Partly

If applicable, is the statistical analysis and its interpretation appropriate?

Yes

Are all the source data underlying the results available to ensure full reproducibility?

Yes

Is the study design appropriate and is the work technically sound?

Yes

Are the conclusions drawn adequately supported by the results?

Partly

Are sufficient details of methods and analysis provided to allow replication by others?

Partly

Reviewer Expertise:

Insect pest management

I confirm that I have read this submission and believe that I have an appropriate level of expertise to state that I do not consider it to be of an acceptable scientific standard, for reasons outlined above.

# Response

**Published:** 2023-11-02

**Authors:** C. A. Zuniga-Gonzalez

**Affiliations:** Agroecology. ECAV. CIByCC., National Autonomous University of Nicaragua, Leon. Bioeconomy and Climate Change Research Center., Leon, Leon, Nicaragua

Reviewver 1  31 Oct 2023 | for Version 2 NOT APPROVED

 We appreciate the comments provided by the reviewer 1. These are the improvements indicated by the reviewer and presenting a new version. We very respectfully apologize for the non-correct placement of Figures 2 and 3, and for the words siuna, which was corrected to sunia.  The improvements we are working on are:

 info_outline

 When referring to a species for the first time in the title as well as in the main text we use the full genus name.

 Title: Write Spodoptera sunia in place of S. sunia

 Background: Write Spodoptera sunia in place of S. sunia

**Response to reviewer**

 Was enhanced it.

 The manuscript needs editing for proper use of English grammar. For instance, I am rewriting the abstract as given below:

 "Methods: The experiment consisted of three treatments and three repetitions involving 30 individuals each. Field-collected S. sunia population was reared in the laboratory under semi-controlled conditions of temperature and humidity for three generations. The progeny of the third generation was crossed with the laboratory population and the resulting progeny was observed for growth and reproductive fitness.

 Results: The results revealed that the hybrid progeny (T3) has a sex ratio (F: M) of  0.82, as against 1.22 and 0.76 observed in the field (T1) and lab populations (T2) respectively. The T1 females oviposited 196 egg masses as against 160 and 59 egg masses by T3  and T2 females respectively. The larval growth was more in hybrid progeny with shorter larval duration. However, no variation was observed in the incubation and pupal periods.

 Conclusion: Since the most optimal results were obtained in T2 and T3,  it is recommended to introduce genetic material every six months to maintain a good laboratory population of the insect host under study and better breeding of its natural enemies."

 Pardon me, I can't reframe the MS beyond this point, it is a tedious job. Kindly get the rest edited for English language and grammar, preferably by a researcher.

**Response to reviewer**

 I would like to express our gratitude for your valuable effort. We have revisited the document and made improvements as per your grammar-related suggestions. We appreciate your contributions in enhancing the summary's composition.

 Also, the authors did not incorporate all the previous suggestions eg. S. siuna is used again in place of S. sunia and limiting two digits after the whole number. eg. 0.4288 mg. It should be 0.43 mg. Also am seeing the same wrongly placed graphs.

**Response to reviewer**

 We have incorporated S sunia as you suggested and have enhanced the graphics by revisiting your previous observation.  We also reviewed the numbers, correcting them to two digits as you suggested. We appreciate your observation in this regard.  The first was in the back review: “Response: Thanks for the observation, we will correct the new version. The problem is that the figures are changed in the place of Figure 2 is Figure 3, and in the place of Figure 3 is Figure 2.”.  Now I am going to make sure to do this in the next version.

 Bst Rgs

 Authors.

# Reviewer report: Reviewer response for version 2

**DOI:** 10.5256/f1000research.154183.r213968

**Published:** 2023-10-31

**Authors:** Hend Omar Mohamed

**Affiliations:** 1 Biological Control Research Department, Plant Protection Research Institute, Agricultural Research Center, Giza, Egypt

**General comment:**

 This study is very important and vital for readers interesting with biological control for improving the host population fitness and hence the biocontrol agent.

 All comments and corrections are highlighted in the article as: 

**Title:** full name of *Spodoptera* *sinus* must write completed without abbreviation in the title and abstract for the first time used because no one can understand what does *S. *mean? Also, Order and Family are not written in italic anyway (Lepidoptera: Noctuidae).

**Abstract**

*Results: *

- In the adult stages T1 and T2 it was 12 days and for T3 10 days please replaced to: In the adult stage, it was 12 days for T1 and T2, but 10 days for T3 was recorded.
- In the egg stage in the three treatments, it was three days replaced with: The egg stage for the three treatments was lasted 3 days...... 

*Conclusions:* ****add *S. sunia* larvae instead larvae of the species under study.

**Introduction:** this section is written well but needs minor corrections as:

- Rearrange the references in all text with the same format either alphabetical or year.
- Transfer this sentence to the end of introduction section (it is an appropriate place): "Ultimately, the subject of study becomes important given the need to study these parameters and the performance of the species *S. sunia* with the introduction of new natural genetic material (cross-species) to improve breeding and maintain optimal parameters ( Raj *et al.*, 2022; Rizo, 1994; Rizo *et al.,* 1994; Passoa, 1991)".
- Replace a biological controller to: a biological control agent. The study to this study. 

**Methods: **this section is clear and written well.

- Delete this sentence: The type of research was experimental and the word species repeated more times.

**Results and discussions:  **

- **'**pre-pupa instar' replaced to 'pre-pupal stage'.
- The legend of Figures 2 and 3 is not correct and un-appropriate. Fig.2 not average weight of larvae? It is developmental duration of each stage of *S. sunia. *
- Fig. 3 is average weight not number of days? Please correct it carefully and the legend color in the graph is not the same of actual line color.
- 'Lose weight to enter the pre-pupa stage' replaced to, 'lose its weight to enter the pre-pupal stage'.
- '0.3018 mg at 20 days in the T2 treatment' instead of, 'the T _2_ treatment a weight of 0.3018 mg at 20 days'. also, 'gr' to 'g.'
- Table 4 legend: 'in different treatments' not 'by treatment'.
- The weight **in mg** repeated more times in all text of the article and it is mentioned previously in the methodology sections so write only the weight without in mg.

*weight and sex of pupae section:*

- '34 are male pupae and 26 female pupae' replaced to '34 males and 26 females'.
- "Treatment T _3_ obtained a total of 60 pupae, 28 females and 32 males for a sex ratio of 0.8:1" - This result is related to treatment T1 not T3, correct it.

*Life cycle:* ****Not Figure 3 but was Figure 2 about average days of each development.

**Conclusions: **

- Replace 'The highest average larval weight obtained from the species *S. sunia* obtained was T _3_ with 0.4288 mg', to 'The highest average larval weight of *S. sunia o*btained from T3 was 0.4288 mg.'
- "of the species *S. sunia" *(delete it) *.*
- *'*the reproduction of the insects of the populations *S. siuna' *replaced to, 'the reproduction of *S. siuna'*.

**Finally, I read and revise this manuscript carefully based on our knowledge and experience** **and hence i confirm that it is of an acceptable partially, however it need some revision and correction before indexing.**

Is the work clearly and accurately presented and does it cite the current literature?

Yes

If applicable, is the statistical analysis and its interpretation appropriate?

Yes

Are all the source data underlying the results available to ensure full reproducibility?

Yes

Is the study design appropriate and is the work technically sound?

Yes

Are the conclusions drawn adequately supported by the results?

Yes

Are sufficient details of methods and analysis provided to allow replication by others?

Yes

Reviewer Expertise:

Biological Control, Integrated pest management and Economic Entomology.

I confirm that I have read this submission and believe that I have an appropriate level of expertise to confirm that it is of an acceptable scientific standard, however I have significant reservations, as outlined above.

# Response

**Published:** 2023-11-02

**Authors:** C. A. Zuniga-Gonzalez

**Affiliations:** Agroecology. ECAV. CIByCC., National Autonomous University of Nicaragua, Leon. Bioeconomy and Climate Change Research Center., Leon, Leon, Nicaragua

Reviewer 2

 General comment:

 This study is very important and vital for readers interesting with biological control for improving the host population fitness and hence the biocontrol agent.

 All comments and corrections are highlighted in the article as:

 Title: full name of Spodoptera sinus must write completed without abbreviation in the title and abstract for the first time used because no one can understand what does S. mean? Also, Order and Family are not written in italic anyway (Lepidoptera: Noctuidae).

**Response to reviewer:**

 Thank you for your insightful comment. We truly believe that this study holds significant importance in the context of biological control, as it contributes to the enhancement of host population fitness and, consequently, the efficacy of biocontrol agents. Your recognition of the study's significance is greatly appreciated.   The observation to the Title was corrected.

 Abstract

 Results:

 In the adult stages T1 and T2 it was 12 days and for T3 10 days please replaced to: In the adult stage, it was 12 days for T1 and T2, but 10 days for T3 was recorded.

  In the egg stage in the three treatments, it was three days replaced with: The egg stage for the three treatments was lasted 3 days.

 Conclusions: add S. sunia larvae instead larvae of the species under study.

**Response to reviewer**

 Dear reviewer, we appreciate your valuable contribution with this observation, however we have adjusted the summary with the contributions of reviewer 1, which coincide with his comments.

 Introduction: this section is written well but needs minor corrections as:

 Rearrange the references in all text with the same format either alphabetical or year.

  Response to reviewer

 Dear reviewer, it was made. In general, all the sections were revised and enhanced.

 Transfer this sentence to the end of introduction section (it is an appropriate place): "Ultimately, the subject of study becomes important given the need to study these parameters and the performance of the species S. sunia with the introduction of new natural genetic material (cross-species) to improve breeding and maintain optimal parameters (Raj et al., 2022; Rizo, 1994; Rizo et al., 1994; Passoa, 1991)".

  Replace a biological controller to: a biological control agent. The study to this study.

**Response to reviewer**

 Dear reviewer, thanks for this observation. It has been made.

 Methods: this section is clear and written well.

 Delete this sentence: The type of research was experimental and the word species repeated more times.

 Response to reviewer

 Dear reviewer thanks for this observation. It has been eliminated.

 Results and discussions: 

 'pre-pupa instar' replaced to 'pre-pupal stage'.

**Response to reviewer**

 Has been corrected.

 The legend of Figures 2 and 3 is not correct and un-appropriate. Fig.2 not average weight of larvae? It is developmental duration of each stage of S. sunia.

  Fig. 3 is average weight not number of days? Please correct it carefully and the legend color in the graph is not the same of actual line color.

**Response to reviewer**

 Dear reviewer thank for this observation. It is correct; the problem was that the figure 2 and 3 inverted are. Has been corrected it.

  'Lose weight to enter the pre-pupa stage' replaced to, 'lose its weight to enter the pre-pupal stage'.

  '0.3018 mg at 20 days in the T2 treatment' instead of, 'the T2 treatment a weight of 0.3018 mg at 20 days'. also, 'gr' to 'g.'

**Response to reviewer**

 Dear reviewer has been corrected it.   Regarding “also, 'gr' to 'g.'” it is ‘mg’.

 Table 4 legend: 'in different treatments' not 'by treatment'.

 The weight in mg repeated more times in all text of the article and it is mentioned previously in the methodology sections so write only the weight without in mg.

**Response to reviewer**

 Ok.  However, I would like to keep it because it is an observation from other reviewers.

 weight and sex of pupae section:

 '34 are male pupae and 26 female pupae' replaced to '34 males and 26 females'.

 "Treatment T3 obtained a total of 60 pupae, 28 females and 32 males for a sex ratio of 0.8:1" - This result is related to treatment T1 not T3, correct it.

 Life cycle: Not Figure 3 but was Figure 2 about average days of each development.

**Response to reviewer**

 Dear reviewer thanks for this observation. Has been corrected it.  The Figure 3 was change for the Figure 2.

 Conclusions:

 Replace 'The highest average larval weight obtained from the species S. sunia obtained was T3 with 0.43 mg', to 'The highest average larval weight of S. sunia obtained from T3 was 0.4288 mg.'

  "of the species S. sunia" (delete it).

  'the reproduction of the insects of the populations S. siuna' replaced to, 'the reproduction of S. suna'.

 Finally, I read and revise this manuscript carefully based on our knowledge and experience and hence i confirm that it is of an acceptable partially, however it need some revision and correction before indexing.

**Response to reviewer**

 Dear reviewer, I thank you for your great contribution and improvement to the quality of the article. We have made the indicated corrections.

 Bst Rgs

 Authors

# Reviewer report: Reviewer response for version 1

**DOI:** 10.5256/f1000research.141849.r178907

**Published:** 2023-07-19

**Authors:** SB Suby

**Affiliations:** 1 ICAR-Indian Institute of Maize Research, Ludhiana, India

The study proved/concluded periodical crossing laboratory population with field population would increase fitness in *Spodoptera sunia.*

**General comment: **After first use of *Spodoptera sunia, *use abbreviated form *S. sunia*.

**Abstract: **Three treatments with 30 individuals and three repetitions. Does it mean 'three treatments with and three repetitions involving 30 individuals each'?

**Title: **The word cross-species literally mean inter-species, thus a misnomer in this study. It shall be replaced with a suitable title like 'periodical crossing of laboratory population with natural population would improve fitness in Spodoptera sunia'. Crossing populations is appropriate in place of 'crossing specimens'.

 Delete the phrase 'It is important to clarify that'

**Methods:** The term 'pre-experimental' will confuse the reader. It is proper experiment only.

 The number of males and females per egg laying cage (replication) should be mentioned in methods.

**Results and discussion:** *Spodoptera **siuna*** (spell check the entire document).leathery worm **donut (donut?)**

 Figure 2 represents duration of stages (Y axis- days in stadium), but it is captioned as weight. Figure 3 is average weight, but titled as number of days. The legend colour given in the graph is different from actual line colour in graph.

 Three digits are enough for fractions eg.0.429 mg in place of 0.4288 mg.

 The egg mass and no. of eggs **per female **should also be mentioned. This should be discussed in comparison with fecundity of S.sunia reported earlier.

**Conclusion: **Closing of populations, not species. The conclusion must focus on periodical crossing and the other statements given on nutrition and environment must be supported by quoting previous studies.

Is the work clearly and accurately presented and does it cite the current literature?

Partly

If applicable, is the statistical analysis and its interpretation appropriate?

Yes

Are all the source data underlying the results available to ensure full reproducibility?

Yes

Is the study design appropriate and is the work technically sound?

Yes

Are the conclusions drawn adequately supported by the results?

Partly

Are sufficient details of methods and analysis provided to allow replication by others?

Partly

Reviewer Expertise:

Insect pest management

I confirm that I have read this submission and believe that I have an appropriate level of expertise to confirm that it is of an acceptable scientific standard, however I have significant reservations, as outlined above.

# Response

**Published:** 2023-08-03

**Authors:** C. A. Zuniga-Gonzalez

**Affiliations:** Agroecology. ECAV. CIByCC., National Autonomous University of Nicaragua, Leon. Bioeconomy and Climate Change Research Center., Leon, Leon, Nicaragua

Response to the reviewer: We appreciate the comments provided by the reviewer. We are working on making the improvements indicated by the reviewer and presenting a new version.

 The improvements we are working on are:

 [1] General comment: After first use of *Spodoptera sunia*, use abbreviated form *S. sunia*.

 Response: We appreciate this observation, if we are correcting it.

 Abstract: Three treatments with 30 individuals and three repetitions. Does it mean 'three treatments with and three repetitions involving 30 individuals each'?

 Response: Dear reviewer, we confirm that it is so.

 [2] Title: The word cross-species literally mean inter-species, thus a misnomer in this study. It shall be replaced with a suitable title like 'periodical crossing of laboratory population with natural population would improve fitness in *Spodoptera sunia*'. Crossing populations is appropriate in place of 'crossing specimens'.

 Response: Dear reviewer, we appreciate your observation, the team agrees and the title change will proceed.

 [3] Delete the phrase 'It is important to clarify that'

 Response: Ok dear it was eliminated

 Methods: The term 'pre-experimental' will confuse the reader. It is proper experiment only.

 Response: Ok thanks for this observation

 [4] The number of males and females per egg laying cage (replication) should be mentioned in methods.

 Response: Thanks. Agreed, the improvement will be made.

 [5] Results and discussion: *Spodoptera siuna* (spell check the entire document).leathery worm donut (donut?)

 Response We thank you for this review, it will be going through the review of a native to improve the spelling it indicates. What is indicated throughout the document will be improved in the next version.

 [6] Figure 2 represents duration of stages (Y axis- days in stadium), but it is captioned as weight.

 Figure 3 is average weight, but titled as number of days. The legend colour given in the graph is different from actual line colour in graph.

 Response: Thanks for the observation, we will make the correction in the new version.

 Three digits are enough for fractions eg.0.429 mg in place of 0.4288 mg.

 Response. Ok thanks for this observation. It will be made.

 [7] The egg mass and no. of eggs per female should also be mentioned. This should be discussed in comparison with fecundity of *S.sunia* reported earlier.

 Response: Thank you for the observation, we confirm that we fully agree and we will work on that improvement.

 [8] Conclusion: Closing of populations, not species. The conclusion must focus on periodical crossing and the other statements given on nutrition and environment must be supported by quoting previous studies.

 Response: Thank you for the observation, we confirm that we fully agree and we will work on that improvement.
